# Effect of Laser Scanning Speed on Microstructure and Properties of Laser Cladding NiAlNbTiV High-Entropy Coatings

**DOI:** 10.3390/ma18174076

**Published:** 2025-08-31

**Authors:** Huan Yan, Shuangli Lu, Lei Li, Wen Huang, Chen Liang

**Affiliations:** 1 School of Materials, Shanghai Dianji University, Shanghai 201306, China; 17333636009@163.com (H.Y.); huangwen0620@163.com (W.H.); 16605145518@163.com (C.L.); 2 School of Mechanical Engineering, Shanghai Dianji University, Shanghai 201306, China; 3 School of Aeronautics, Shanghai Dianji University, Shanghai 201306, China

**Keywords:** high-entropy alloy, microstructure, friction and wear resistance, scanning speed

## Abstract

High-entropy alloys (HEAs) exhibit superior properties for extreme environments, yet the effects of laser scanning speed on the microstructure and performance of laser-clad NiAlNbTiV HEA coatings remain unclear. This study systematically investigates NiAlNbTiV coatings on 316 stainless steel fabricated at scanning speeds of 800–1100 mm/min via laser cladding. Characterizations via XRD, SEM/EDS, microhardness testing, high-temperature wear testing, and electrochemical measurements reveal that increasing scanning speed enhances the cooling rate, promoting γ-(Ni, Fe) solid solution formation, intensifying TiV peaks, and reducing Fe-Nb intermetallics. Higher speeds refine grains and needle-like crystal distributions but introduce point defects and cracks at 1100 mm/min. Microhardness decreases from 606.2 HV (800 mm/min) to 522.4 HV (1100 mm/min). The 800 mm/min coating shows optimal wear resistance (wear volume: 0.0117 mm^3^) due to dense eutectic hard phases, while higher speeds degrade wear performance via increased defects. Corrosion resistance follows a non-linear trend, with the 900 mm/min coating achieving the lowest corrosion current density (1.656 μA·cm^−2^) due to fine grains and minimal defects. This work provides parametric optimization guidance for laser-clad HEA coatings in extreme-condition engineering applications.

## 1. Introduction

High-entropy alloys (HEAs) have emerged as critical materials in industrial applications. As the aerospace, energy, automotive, and defense industries continue to evolve, the demand for advanced alloy materials has grown significantly [1]. HEAs exhibit outstanding high-temperature strength, corrosion resistance, and oxidation resistance, making them ideal candidates for extreme environments such as aircraft engines, gas turbines, chemical reactors, and nuclear facilities [2,3,4,5,6]. The NiAlNbTiV HEA demonstrates superior hardness, exceptionally high-temperature performance, corrosion resistance, wear resistance, and relative stability [7,8,9], while exposure of the NiAlNbTiV HEA to high-temperature and high-pressure conditions results in significant surface wear, which degrades its mechanical properties and indirectly raises operational costs [1,10,11].

316 stainless steel is widely utilized in industries such as chemical engineering and petroleum production due to its versatility [12]. It maintains stability at elevated temperatures and possesses good machinability [13]. However, its single-phase austenitic structure leads to relatively low hardness and poor wear resistance [14]. To mitigate these limitations, an HEA coating of NiAlNbTiV was applied to the surface of stainless steel components using laser cladding technology for repair and strengthening. This application enhances surface hardness and wear resistance, thereby extending component service life and improving reliability. Laser cladding is an advanced surface engineering technology that employs a high-energy laser beam to melt metallic materials (powder or wire), forming a metallurgical bond with the substrate surface [15,16,17,18]. Through precise control of laser energy, high-performance materials are deposited layer-wise onto the substrate surface, forming dense, uniform, and strengthened coatings that enhance wear, corrosion, high-temperature, and fatigue resistance; these coatings may also serve to restore damaged components [19,20,21,22]. This technique offers high precision, low heat input, improved bond strength, and compositional flexibility, making it widely applicable in high-end manufacturing, remanufacturing, and additive manufacturing [23,24,25,26,27].

Despite favorable mechanical properties, laser-clad coatings invariably develop defects and residual stresses due to the rapid thermomechanical cycling inherent in the process [28,29,30]. To overcome these inherent process limitations, parametric optimization has been widely investigated as a primary approach for coating quality. Linear energy density (E), a key process parameter that integrates multiple operational variables, plays a fundamental role in process control. This critical parameter, expressed in J/mm^2^, is mathematically defined as follows:

*E* = *P*/(*V* ∗ *d*) (1)

In Equation (1), *P* denotes laser power (W), *V* represents scanning speed (mm/s), and *d* corresponds to beam diameter (mm). Jiao et al. [31] demonstrated the laser-clad deposition of the T15M high-speed steel alloy onto Q235 substrates, revealing that increasing scanning speed to 5 mm/s promoted grain-boundary carbide precipitation while introducing porosity, microcracks, and inclusions. Qiao’s work [32] established that higher scanning speeds (up to 8 mm/s) suppress dendritic growth due to enhanced cooling rates (>10^3^ K/s), thereby modifying microhardness gradients and corrosion behavior. Xu et al. [33] fabricated Ti6Al4V-based composites with 90 wt.% TiC-10 wt.% Ni60 reinforcement via laser cladding, showing an inverse correlation between scanning speed and coating hardness, with corresponding wear rates increasing by 40%. TiC reinforcement reduced the specific wear rate by 65% compared to monolithic Ni60. Yuan’s [34] revealed that high-speed laser cladding (HSLC) enhances deposition efficiency by three to five times relative to conventional processes, while expanding viable applications through reduced thermal budgets. HSLC-processed coatings show a positive correlation with cladding speed in the 8–15 m/min range, exhibiting 25–30% lower wear rates and 1.5× higher polarization resistance compared to conventional counterparts. Zhang et al. [35] systematically compared CoCrFeMnNi HEA coatings produced via HSLC and conventional methods, revealing that HSLC enables 1.8-times grain refinement, limits dilution to <5%, and enhances microhardness with 50% lower corrosion current density. Meanwhile, Xu Z et al. [36] found during laser cladding of IN718 that the scanning speed was a very significant factor affecting the maximum load value. Recently, M et al.’s [37] research found that as the scanning speed increases, the spacing between primary and secondary dendrite arms decreases by approximately 14% and 17%, respectively.

Despite the significant advantages of HEAs and laser cladding technology, a critical gap persists in knowledge of the influence of scanning speed on the microstructure and wear resistance of NiAlNbTiV HEA coatings. The influence of specific scanning speeds on microstructural changes, grain refinement, high-temperature wear resistance, friction and wear mechanisms, and high-temperature corrosion resistance in HEA coatings has been largely overlooked by most researchers; these aspects remain to be fully elucidated. Therefore, in order to make up for the lack of research regarding this aspect, this study aims to systematically explore the influence of varying scanning speeds on optimizing process parameters, enhancing the performance of the coatings at high temperatures, and elucidating structure–property relationships. The findings are intended to provide a theoretical foundation and technical guidance for advancing the engineering applications of HEAs under extreme operating conditions.

## 2. Experimental Procedure

**Figure 1:**
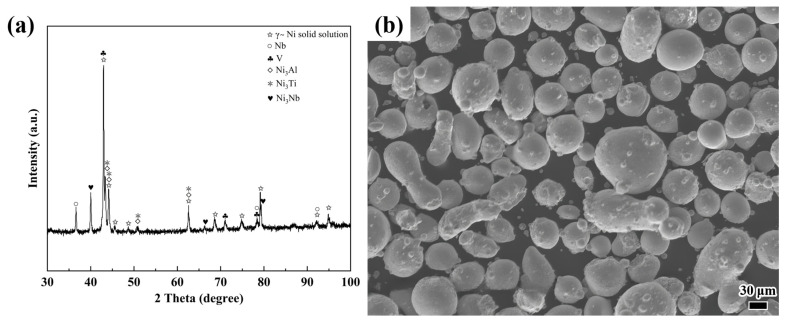
Microstructure and phase composition of NiAlNbTiV powder: (**a**) X-ray diffraction (XRD) pattern; (**b**) surface morphology.

In this study, 316 stainless steel plates (100 mm × 100 mm × 10 mm) were employed as the substrate for laser cladding. Prior to cladding, the surface of the substrate was sanded with 2000-grit sandpaper to remove the surface oxide layer and ultrasonically cleaned using acetone. The experimental powder was NiAlNbTiV pre-alloyed powder, with an average particle size of approximately 20 to 80 μm. The chemical compositions of the substrate and powder are presented in Table 1, while the morphology and phase analysis of the NiAlNbTiV powder are shown in Figure 1. As shown in Figure 1a, the powder alloy is composed of three metal compounds, all predominantly Ni-based, owing to the relatively high Ni content (Table 1). This alloy powder is spheroidized to enhance its flow characteristics during the laser cladding process and to achieve a more uniform coating composition. The primary focus of this study is the NiAlNbTiV coating; hence, the microstructure of the substrate (316L steel) is not examined.

**Figure 2:**
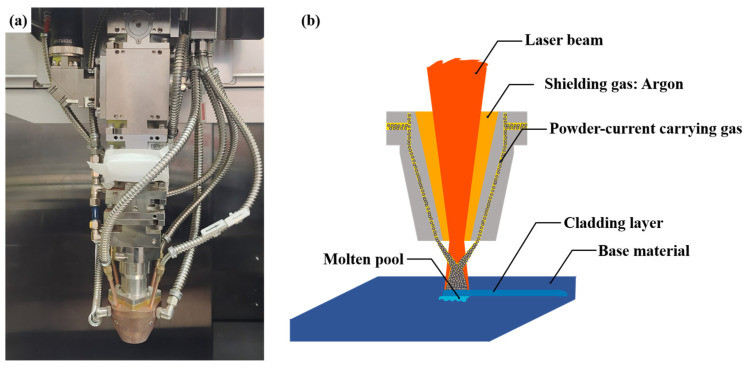
Schematic diagram of the laser and coaxial pneumatic powder feeding system: (**a**) RG-LCD-50R-40F laser; (**b**) coaxial pneumatic powder feeding system schematic.

An RG-LCD-50R-40F laser (Nanjing Zhongke Yuchen Laser Technology Co., LTD, Nanjing, China) with a wavelength of 1064 nm and a spot diameter of 3.0 mm was employed as the light source. The process utilized coaxial pneumatic powder feeding, as illustrated in Figure 2. Ultra-high-purity argon (99.99% purity) was used as the powder carrier gas, while argon of equivalent purity served as the shielding gas at a flow rate of 20 L/min. The laser melting process parameters were as follows: power (*P*) = 1500 W, powder feed rate = 1.5 g/min, lap rate = 50%, and scanning speeds = 800 mm/min, 900 mm/min, 1000 mm/min, and 1100 mm/min. The size of the coating was 40 mm ∗ 30 mm, the length of the laser cladding track was 40 mm, and the quantity was 20.

Following the laser cladding process, the specimens were sectioned into samples measuring 10 mm × 10 mm using wire electrical discharge machining. This approach facilitates a clearer observation of a coating’s cross-section and enables the conduction of subsequent tests.

The samples underwent a series of surface preparation steps, which included rough grinding, fine grinding, rough polishing, fine polishing, and etching. For the etching process, aqua regia was used. This is a freshly prepared mixture of concentrated hydrochloric acid and concentrated nitric acid in a volume ratio of 3:1. The microstructure and elemental distribution of the cladding coating were characterized using a scanning electron microscope (SEM; S-3400N, Hitachi, Tokyo, Japan) combined with an energy-dispersive spectrometer (EDS; S-3400N, Hitachi, Tokyo, Japan), operated at an acceleration voltage of 25 kV.

Phase analysis of the NiAlNbTiV cladding coating was conducted using an X-ray diffractometer (XRD; D8-ADVANCE, Bruker, MA, USA). The diffraction angle ranged from 30° to 100°, with a scanning rate of 6°/min. Cu-Kα radiation was employed at a working voltage of 40 kV and a current of 40 mA.

The microhardness distribution of the coating was assessed using a Vickers microhardness tester (FM-700/SVDM4R, FUTURE-TECH, Tokyo, Japan). Hardness measurements were taken at intervals of 0.1 mm from the coating surface down to the substrate, with all indentation experiments conducted at room temperature. An applied load of 1 kg was used, with a holding time of 10 s. To enhance data accuracy, all microhardness values reported in this study represent the averages of three independent measurements obtained from the same sample.

The wear resistance of the coating was assessed using a multifunctional friction and wear tester (HT-10000, Lanzhou, China), and a coefficient of friction (COF) curve was recorded. The test setup included a circular friction and wear test conducted under the following conditions: a load of 20 N, a rotational speed of 200 r/min, a rotation radius of 5 mm, a test duration of 10 min, and a test temperature of 800 °C, with Si_3_N_4_ as the friction pair. A laser confocal microscope (LEXT-OLS5100, Olympus, Tokyo, Japan) was employed to inspect the surface and 3D morphology of the coating, as well as to quantify the wear volume following friction and wear tests. An electronic scale was also used to measure the wear mass loss. The coefficient of friction (COF) mentioned above is defined as the ratio of the friction force between two contacting surfaces to the normal force exerted between them [38]. It is a dimensionless physical quantity that characterizes the frictional resistance between objects, typically ranging from 0 to 1. A higher COF signifies greater frictional resistance, whereas a lower COF indicates reduced frictional resistance.

The corrosion resistance of the cladding was assessed using an electrochemical workstation (AUTOLAB-PGSTAT302N, Metrohm, Switzerland). A saturated calomel electrode (SCE) was used as the reference electrode, and a platinum (Pt) electrode served as the auxiliary electrode. The treated sample was immersed in a 3.5 wt.% NaCl solution and kept at 80 °C in a thermostatic water bath (Longyue SYG-2, Changzhou, China). Once the open-circuit potential stabilized for 30 min, electrochemical impedance spectroscopy (EIS) and potentiodynamic polarization measurements were carried out. The resulting anodic polarization and impedance curves were then analyzed using the instrument’s dedicated software.

## 3. Results and Discussion

### 3.1. Phase Analysis

**Figure 3:**
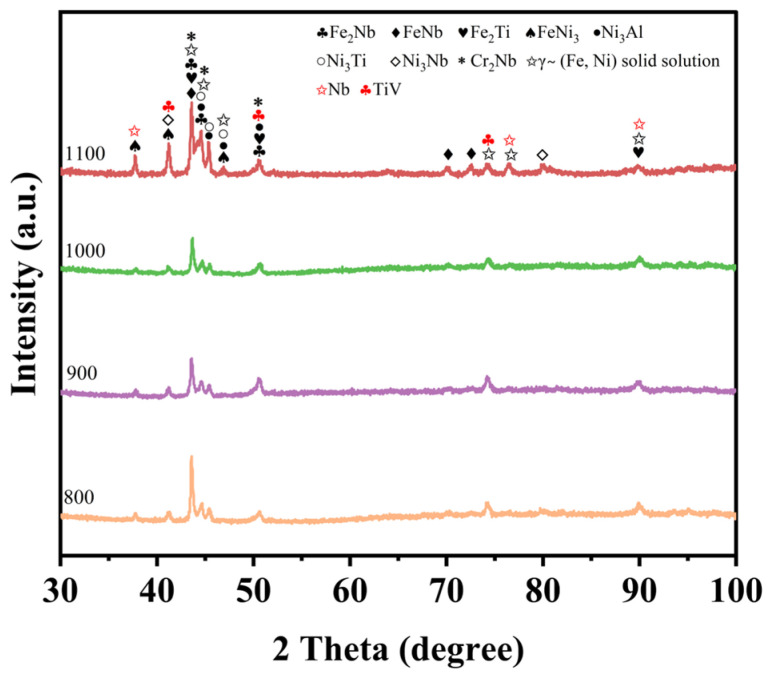
XRD patterns of laser-clad layers as a function of scanning speed.

Figure 3 presents the X-ray diffraction (XRD) patterns of the NiAlNbTiV cladding coating at different laser scanning speeds. The results indicate that the NiAlNbTiV coating consists of a γ-(Ni Fe) solid solution and a variety of metal compounds.

As depicted in Figure 3, the XRD pattern of the laser-clad NiAlNbTiV coating changes with increasing scanning speed. As the scanning speed increases, the intensity of the TiV peaks rises. This is attributed to insufficient heating power at high scanning speeds, which limits the diffusion of V into the lattice [31]. Additionally, an increase in the austenitic phase is observed as the scanning speed increases from 800 mm/min to 1100 mm/min. Previous studies have confirmed that increasing the scanning speed effectively enhances the cooling rate during solidification [39]. As the scanning rate increases, the substrate dilution rate decreases, leading to a reduction in Fe content, as evidenced by the Fe_2_Nb and FeNb peak intensity in Figure 3. This occurs because at slower scanning speeds, Fe has sufficient time to react with Ni and other elements to form phases such as Ni-Fe solid solutions and Fe-Nb intermetallic compounds. Compared with other XRD curves, the 1100 mm/min XRD curve has four more peaks, with 2θ values ranging from 70° to 85°. This phenomenon occurs because an increase in the scanning rate leads to a corresponding rise in the cooling rate. The rapid cooling limits atomic diffusion, prompting the originally supersaturated solid solution to undergo desolvation decomposition and precipitate a portion of the phase. Concurrently, the dilution rate decreases, resulting in a reduced proportion of Fe elements in the coating, while the proportions of elements such as Ni and Nb increase.

### 3.2. Microstructure Analysis

**Figure 4:**
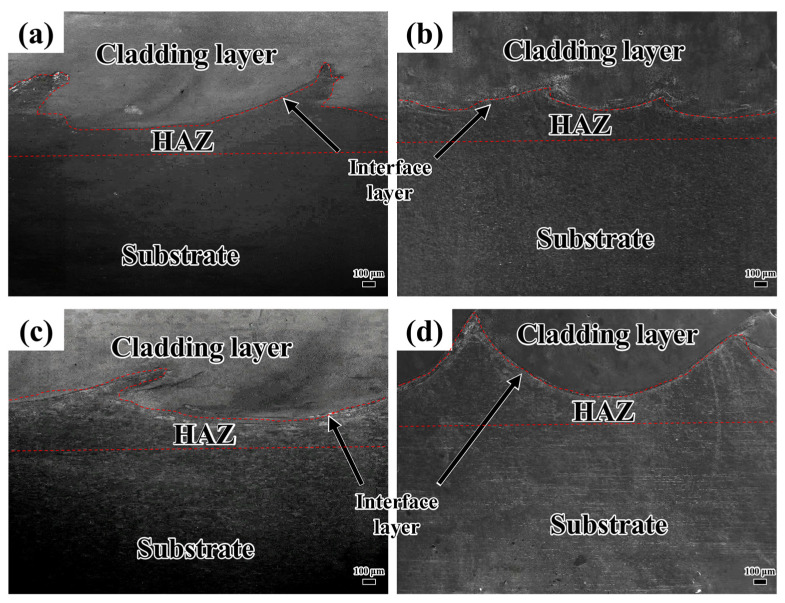
Microstructure at the interface between coating and substrate at different laser scanning speeds: (**a**) 800 mm/min; (**b**) 900 mm/min; (**c**) 1000 mm/min; (**d**) 1100 mm/min.

Figure 4 illustrates the microstructure at the interface between the coating and the substrate for various laser scanning speeds. As shown in Figure 4, no penetrating cracks were observed at the interface between the substrate and the coating. There is an unfilled gap between the coating and the substrate. There are no densely distributed pores near the interface. On the contrary, the pores are linearly distributed along the interface. These results demonstrate that the interface bonding quality of the coatings prepared at various laser scanning speeds is satisfactory.

**Figure 5:**
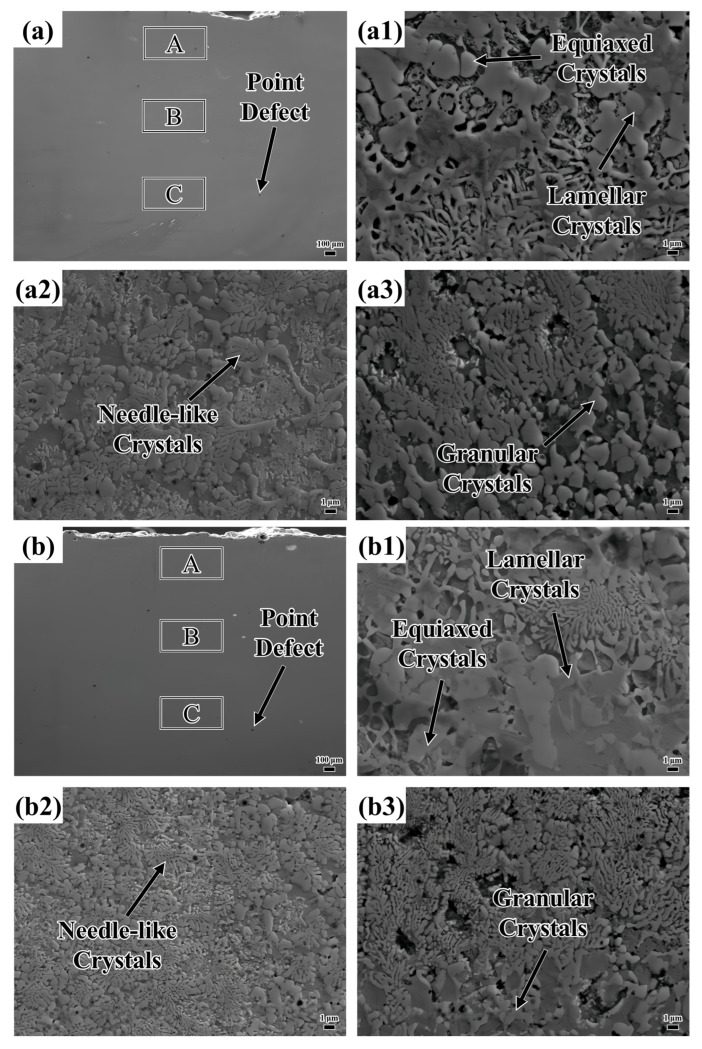

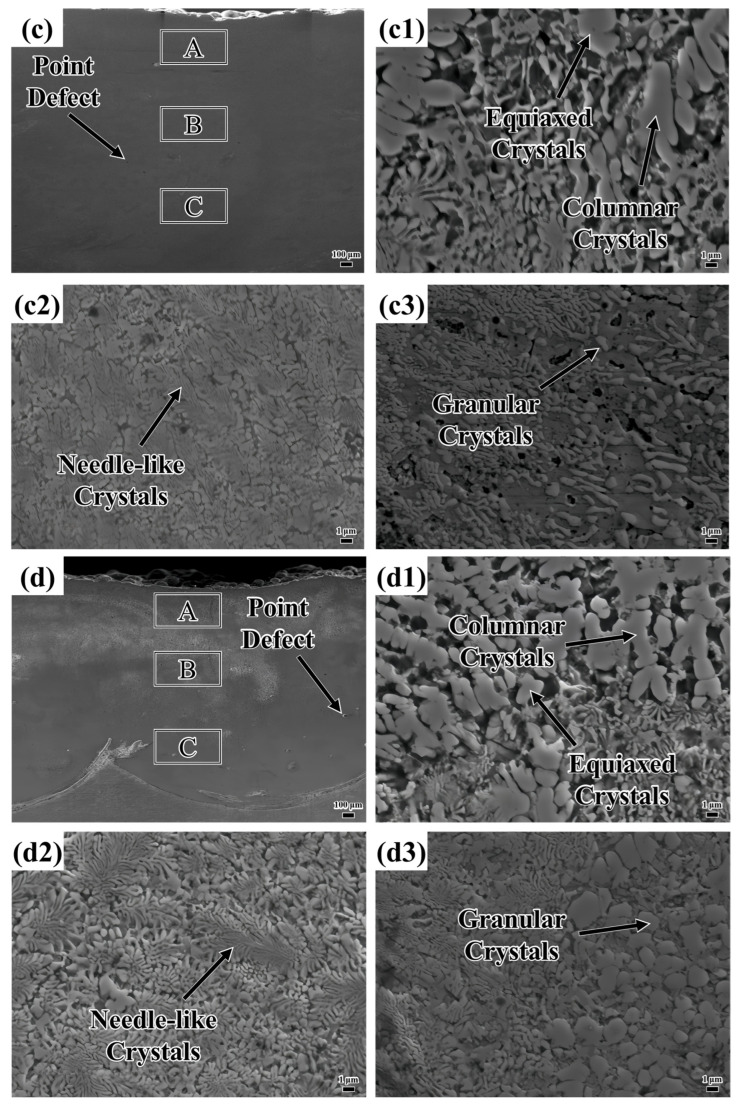
Microstructure of the coating: (**a**–**d**) represent laser coatings with laser scanning speeds of 800 mm/min, 900 mm/min, 1000 mm/min, and 1100 mm/min, respectively. (**a1**–**a3**, **b1**–**b3**, **c1**–**c3**, and **d1**–**d3**) correspond to the regional topography of A, B, and C in Figures (**a**–**d**), respectively.

Figure 5 shows the microstructure of the coating. The defects existing in the layers are marked in Figure 5a–d. The formation of these defects is attributed to the decrease in energy density as the laser scanning speed increases, resulting in insufficient melting of the powder. The powder’s small particle size enhances its adsorption properties, making it prone to attracting substances like nitrogen from the laser processing protective gas [40]. During laser processing, these adsorbed gases are released, leading to pore formation. This is because NiAlNbTiV powder (100–300 μm), due to its fine particle size and active elements, is prone to adsorbing gases and has a very high surface adsorption capacity. In contrast, the defects in other HEAs (for example, WC-enhanced coatings [25]) result from gas capture during the melting process rather than powder adsorption. Table 2 shows the number of coating point defects within 1 mm^2^; the point defects in the coating increase with rising laser scanning speeds.

Illustrated in Figure 5(a1–a3), the top layer of the coating predominantly features lamellar and needle-like crystalline structures. The middle section is primarily characterized by needle-like crystals, interspersed with a modest quantity of granular crystals. In the lower area, needle-like and granular crystals coexist, with the granular crystals progressively evolving into needle-like crystals as the distance from the heat source increases. The uppermost region of the coating is directly subjected to laser energy, with the molten pool surface interfacing with the surrounding protective gas. This interaction leads to an exceptionally high cooling rate. Such rapid solidification restricts solute diffusion (for example, Nb), thereby generating an unbalanced eutectic structure. Eutectics are organized into regular lamellar or fibrous arrangements, creating a pronounced temperature gradient perpendicular to the molten pool surface. This gradient encourages crystal growth in the direction of heat flow. In the central area, which is distant from the molten pool surface, the cooling rate diminishes, allowing for substantial solute diffusion. The decrease in the temperature gradient and the more scattered direction of heat flow result in disordered crystal growth, with local solute enrichment giving rise to needle-like crystals. In proximity to the substrate, heat is rapidly conducted, which further increases the cooling rate. Subsequent laser scanning has a thermal impact on the bottom area, partially melting the crystal structure. A slower cooling rate permits sufficient solute diffusion, leading to the formation of coarse granular crystals. As temperature fluctuations occur, granular crystals partially dissolve in the ensuing thermal cycle. The redistribution and diffusion of the solute gradually erase the regular boundaries, resulting in finer needle-like crystals.

Upon inspection of Figure 5(a1,b1), it is evident that plate-like crystals are present at the top of both layers. However, when the sweep speed was elevated to 1000 mm/min, a small quantity of columnar crystals emerged at the top of the coating, as illustrated in Figure 5(c1). At a sweep speed of 1100 mm/min, the area occupied by the columnar crystals at the top of the coating increased substantially, as shown in Figure 5(d1). Moreover, it is noteworthy that the needle-like crystals in the middle of the coating become more evenly distributed and refined with increasing scanning speed. As the scanning speed goes up, granular crystals tend to transform into needle-like crystals, as depicted in Figure 5(a2–d2). In Figure 5(a3), both granular and needle-like crystals are relatively large in size. As the scanning speed is increased, the sizes of these crystals diminish and their distribution becomes more uniform, as illustrated in Figure 5(b3). Further increasing the scanning speed causes the sizes of the granular and needle-like crystals to decrease even more. However, at this point, their distribution becomes loose and non-uniform, as shown in Figure 5(c3). When the scanning speed reaches 1100 mm/min, the granular and needle-like crystals are uniformly distributed and have a high density, but the size of the granular crystals increases.

Since this alloy does not contain Fe or Cr elements, we tested the mass fractions of different elements on the surface of the coating to reflect from the side the dilution degree of the base material in the coating. The specific results are shown in Table 3. When the sweeping speed increased from 800 mm/min to 1100 mm/min, the Fe and Cr elements in the coating surface also decreased from 34.84 wt.% and 12.25 wt.% to 8.05 wt.% and 2.01 wt.%, respectively.

**Figure 6:**
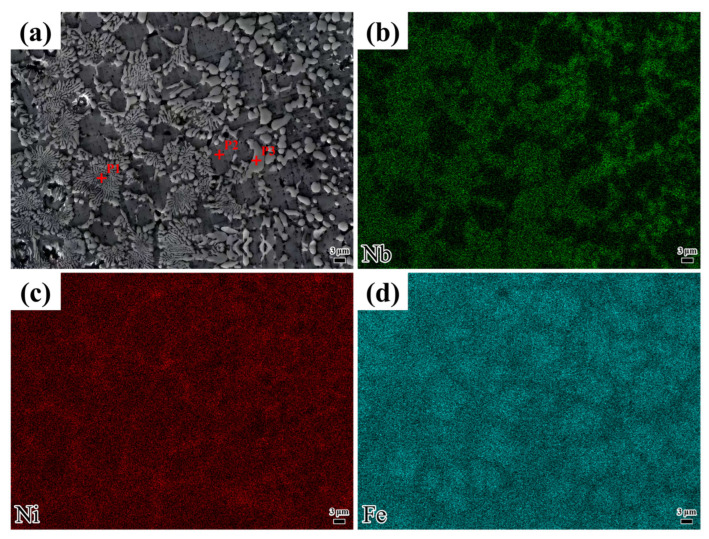
Laser cladding at 1000 mm/min: (**a**) microstructural features; (**b**–**d**) energy-dispersive spectroscopy (EDS) analysis of different microstructures.

Figure 6 displays the microstructure of the coating deposited at a laser scanning speed of 1000 mm/min, along with the distribution of elements in different morphologies. As shown in Table 4, the Ni content is higher than that of Nb. However, Table 4 shows that the P1 position corresponds to a Nb-rich region. Based on XRD analysis, the phase at point P1 is identified as Ni_3_Nb. Point P1 exhibits the highest Fe content, suggesting the potential formation of a γ-Fe solid solution. Point P2 shows very high Fe and Ni contents with very low Nb contents, indicating the possible presence of a γ-(Ni Fe) solid solution. A small amount of unreacted Ni may exist in solid solution form. Point P3, with a high Nb content, may form Nb-rich intermetallic compounds. By integrating the Ni and Nb content data with the XRD results, it can be concluded that the phase may contain Ni_3_Nb. Additionally, the combination of Fe and Nb content data suggests the possible presence of an Fe_2_Nb phase. The Cr contents at P1, P2, and P3 are also relatively significant and may combine with C to form hard, wear-resistant phases.

As depicted in Figure 6c,d, the elements Ni and Nb are predominantly concentrated in the non-eutectic structural regions. Based on the above analysis, the eutectic structure at position P1 is attributed to a non-equilibrium eutectic reaction induced by the rapid solidification characteristic inherent in laser cladding, resulting in a lamellar structure composed of alternating Ni-Nb intermetallic compounds and a γ-(Ni Fe) solid solution. The rapid solidification inherent in laser cladding restricts the full diffusion of solute atoms (e.g., Nb), trapping them at the solidification front and facilitating the development of a non-equilibrium eutectic structure. Additionally, high-melting-point elements like Nb are expelled to interdendritic or grain-boundary regions during solidification, where they combine with elements such as Ni to initiate eutectic reactions. The sufficiently high cooling rate suppresses long-range atomic diffusion, causing the liquid phase to directly transform into a eutectic two-phase structure. The formation mechanism is governed by solute retention, elemental segregation, and rapid cooling.

**Figure 7:**
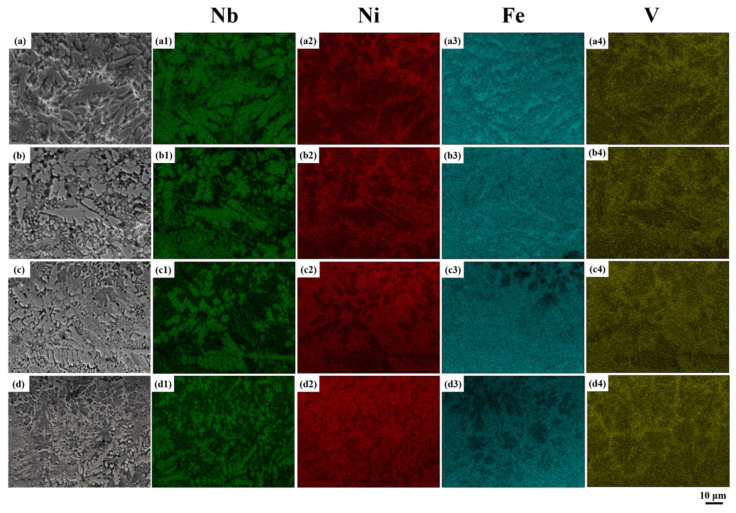
EDS mapping of Nb, Ni, Fe, and V in the upper part of coatings deposited at different laser scanning speeds: (**a**) SEM images (**a1**–**a4**) showing element distribution at V = 800 mm/min; (**b**) SEM images (**b1**–**b4**) showing element distribution at V = 900 mm/min; (**c**) SEM images (**c1**–**c4**) showing element distribution at V = 1000 mm/min; (**d**) SEM images (**d1**–**d4**) showing element distribution at V = 1100 mm/min.

As depicted in Figure 7(a1–a4), Nb and Fe are primarily concentrated in the eutectic structure, while Ni and V display an opposing distribution. An increase in laser scanning speed enhances the cooling rate, leading to a gradual rise in supercooling, as illustrated in Figure 7a–d. This results in the progressive refinement of the eutectic structure in the coating and an increase in Ni content, as observed in Figure 7(a2–d2). Moreover, as the laser scanning speed increases, the interaction time of the laser energy decreases, resulting in a reduced molten pool height. The diminished thermal diffusion between the deposition layer and substrate leads to decreased molten pool depth with increasing scanning speed. The Fe content decreases in non-eutectic regions but remains in eutectic regions, albeit at lower concentrations. Fe preferentially reacts with Nb to form the Fe-Nb intermetallic phase. As shown in Figure 7(a4–d4), with increasing laser scanning speed, pronounced V-rich regions that exhibit an overall network morphology are formed. The V segregation observed is unique to NiAlNbTiV, as V has limited solubility in most HEAs, such as Ni60-based composites [33] or CoCrFeNiW [26], and rarely forms this network structure. This phenomenon arises from the high mobility of V in the alloy during rapid cooling, a behavior that has not been documented in prior studies.

**Figure 8:**
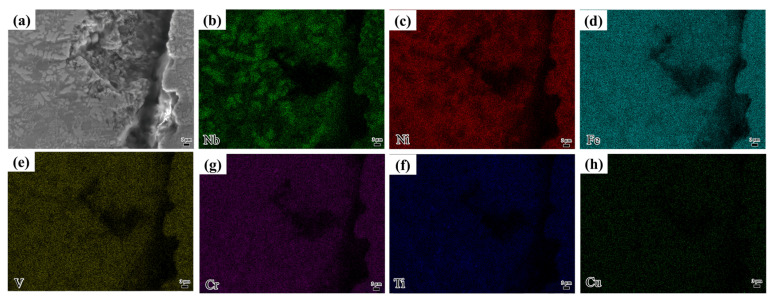
Laser cladding at 1100 mm/min: (**a**) crack morphology; (**b**–**h**) EDS analysis of the crack region.

At a laser scanning speed of 1100 mm/min, cracks were observed in the coating, as depicted in Figure 8. As the laser scanning speed increases, the heat input per unit area decreases, resulting in a reduced molten pool temperature and shorter existence time. The rapid cooling increases the temperature gradient between the coating and substrate, weakening the metallurgical bond and causing residual stresses due to differences in thermal expansion coefficients. These stresses exceed the material’s strength, leading to crack formation. Notably, no elemental enrichment was observed at the crack, as shown in Figure 8b–d. This is in stark contrast to Al-rich HEAs [18], where the segregation of Al at grain boundaries tends to facilitate crack initiation. The superior crack resistance of NiAlNbTiV against segregation-induced failure can be attributed to its uniform Ni matrix and well-balanced elemental diffusion, which are unique material-specific advantages.

### 3.3. Microhardness

**Figure 9:**
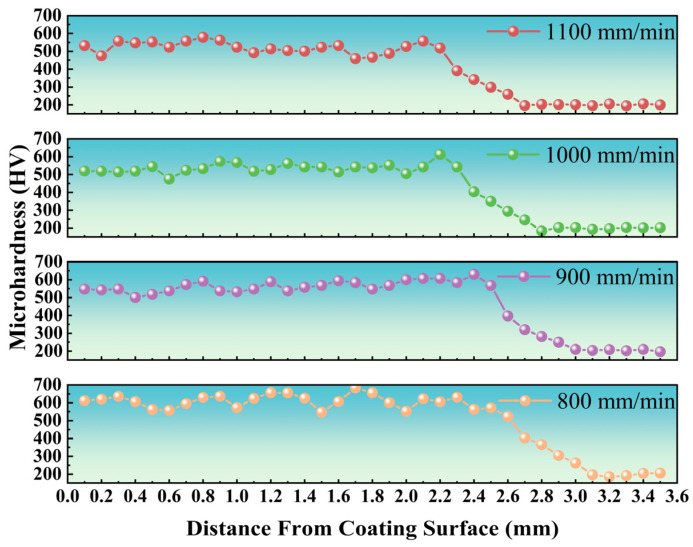
Microhardness distribution of coatings with different laser speeds.

Figure 9 presents the microhardness of coatings at varying laser scanning speeds. The coating hardness depicted in Figure 6 exhibits notable fluctuations, primarily due to the varying hardness values associated with the eutectic structure and the solid solution. As illustrated in Table 5, the coating hardness diminishes as the laser scanning speed increases. Specifically, the average hardness for the coating produced at 800 mm/min is 606.2 HV, whereas the average hardness for the coating produced at 1100 mm/min is 522.4 HV.

At lower laser scanning speeds (e.g., 800 mm/min), extended solute diffusion time promotes the nucleation and growth of a significant volume fraction of hard phases. Intermetallic compounds (e.g., Fe_2_Nb, FeNb, and Ni_3_Nb) play a crucial role in enhancing the material’s macrohardness. However, as the laser scanning speed increases, the solute diffusion time decreases, which could inhibit the precipitation of hard phases and lead to a gradual reduction in macrohardness. At higher scanning speeds, the γ-FeNi solid solution content reaches near saturation, and this will diminish the impact of hard-phase depletion on hardness degradation. Below a critical volume fraction of hard phases, the solid solution strengthening mechanism becomes dominant, stabilizing the macrohardness and mitigating further decreases. Simultaneously, grain refinement approaches a saturation point at higher scanning speeds, enhancing its compensatory effect on hardness retention and reducing the extent of hardness loss. Additionally, elevated residual stresses and dislocation densities generated at high scanning speeds partially offset the negative effects of hard-phase depletion on macrohardness.

### 3.4. Analysis of Friction–Wear Properties

**Figure 10:**
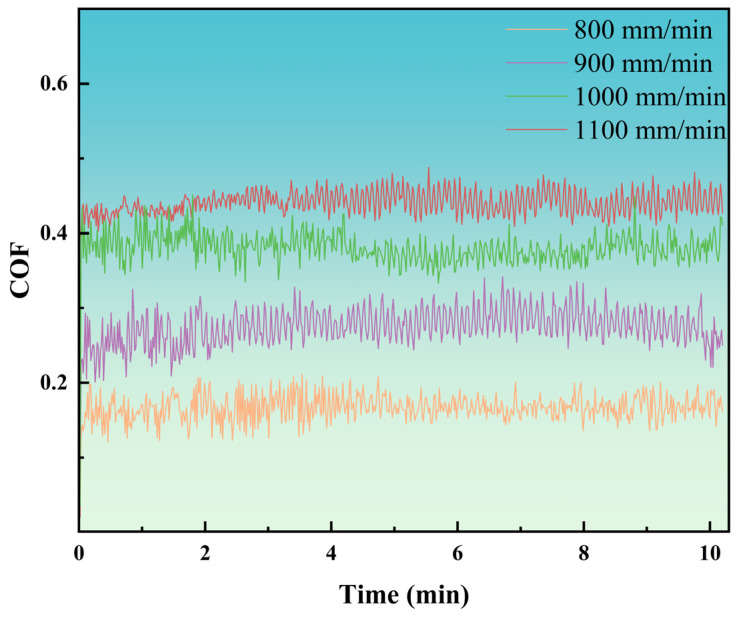
COF of coatings at various laser scanning speeds.

**Figure 11:**
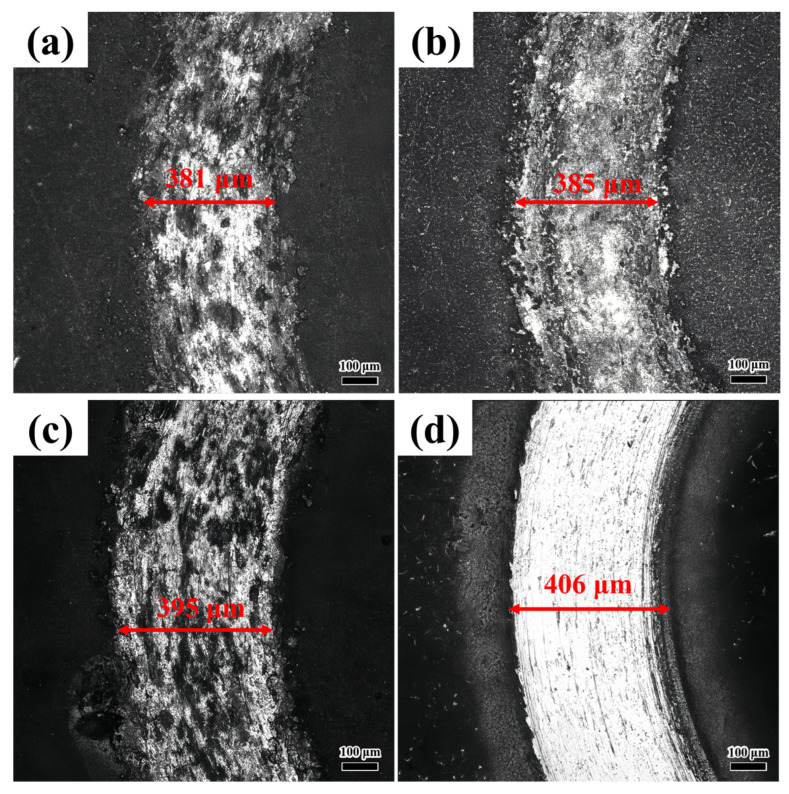
The friction and wear morphologies of coatings at varying laser scanning speeds, as observed via optical microscopy: (**a**) 800 mm/min; (**b**) 900 mm/min; (**c**) 1000 mm/min; (**d**) 1100 mm/min.

Figure 10 shows the COF of the coating at different laser scanning speeds. As shown in Figure 10, the COF rises steadily with an increase in scanning speed. This trend is closely linked to the microhardness of the coating. At a scanning speed of 800 mm/min, the coating demonstrates the highest microhardness (606.2 HV). This elevated hardness enhances the coating’s resistance to plastic deformation when subjected to sliding friction pairs (Si_3_N_4_), thereby reducing the actual contact area between the coating and the substrate. The decreased contact area, in turn, diminishes frictional resistance, leading to the lowest COF. Conversely, when the scanning speed is increased to 1100 mm/min, the microhardness drops to 522.4 HV. The lower hardness makes the coating more susceptible to plastic deformation during sliding, which enlarges the actual contact area and consequently raises the COF, as shown in Figure 11a–d. As the scanning speed increases, the gradual rise in defect density further exacerbates the coefficient of friction (COF). The coating, characterized by minimal porosity and the absence of cracks, exhibits a dense microstructure that effectively resists the ingress of abrasive particles. This dense configuration mitigates localized stress concentrations during the sliding process, thereby restricting material removal and helping to sustain a lower COF.

**Figure 12:**
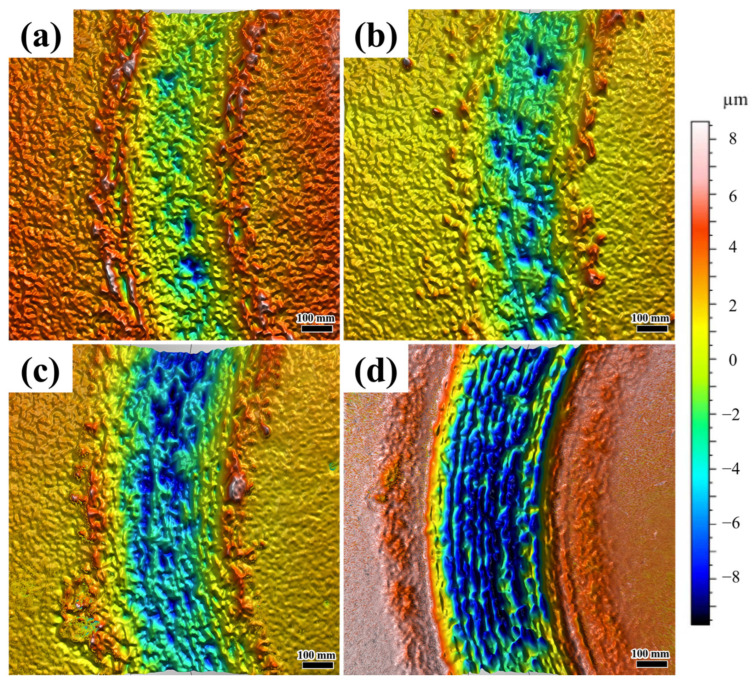
Three-dimensional morphology of friction–wear behavior in coatings under different laser scanning speeds: (**a**) 800 mm/min; (**b**) 900 mm/min; (**c**) 1000 mm/min; (**d**) 1100 mm/min.

Figure 12 presents the 3D morphology of friction–wear behavior in coatings under different laser scanning speeds. At a scanning speed of 800 mm/min, the coating wear trajectory displays a relatively smooth three-dimensional profile with shallow and small grooves. This corresponds to mild abrasive wear (Figure 12a), where the dense eutectic hard phase (Ni_3_Nb and Fe_2_Nb) resists severe material removal, resulting in minimal wear depth and uniform surface damage. In Figure 12b, localized shallow pits are observed, signaling the initiation of adhesive wear. However, the overall surface still maintains a relatively continuous profile. In contrast to the coating produced at 900 mm/min, as shown in Figure 12c, the wear trajectory of the coating deposited at 1000 mm/min demonstrates pronounced deepening and widening, giving rise to distinct deep pits and irregular grooves. This suggests that the increased defect density and the brittle Fe-Nb phase exacerbate fatigue wear. As depicted in Figure 12d, the wear trajectory sustains the most severe damage, with a maximum depth of approximately −8 μm. The surface is characterized by extensive deep pits and interconnected grooves. This observation aligns with the characteristics of severe adhesive wear. The high defect density (28 holes/mm^2^) and the presence of cracks serve as stress concentrators, which expedite material spalling. As indicated in Table 6, the coating produced at a scanning speed of 800 mm/min demonstrates the smallest wear volume (0.0117 mm^3^) and mass loss (0.5089 g), which confirms its superior wear resistance. Conversely, the coating deposited at 1100 mm/min exhibits the largest wear volume (0.0319 mm^3^) and mass loss (1.3895 g).

**Figure 13:**
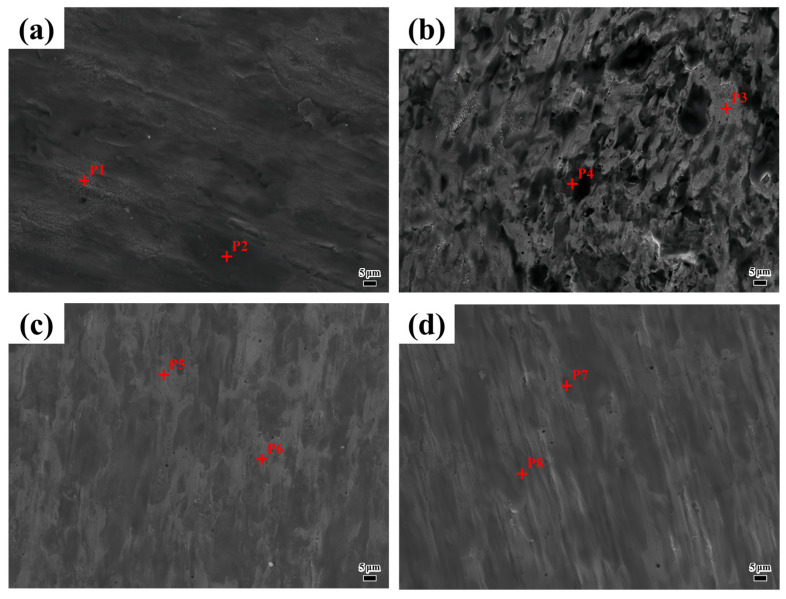
The friction and wear morphology of the coating with different laser scanning speeds and the elemental composition of different morphologies: (**a**) 800 mm/min; (**b**) 900 mm/min; (**c**) 1000 mm/min; (**d**) 1100 mm/min.

Figure 13 shows the friction and wear morphology of the coating with different laser scanning speeds and the elemental composition of different morphologies. Since this friction and wear test was performed in a non-vacuum environment, the oxygen distribution is illustrated in Table 7. The oxygen content in the dark region is substantially higher than in the bright region, suggesting that the dark area may correspond to a metal oxide film. As shown in Figure 13a, the worn surface of the coating exhibits deep grooves and spalling pits, indicating that abrasive wear is the primary wear mechanism, accompanied by localized adhesive wear. The oxygen content is relatively low, and the oxide layer is thin, such that it cannot effectively prevent direct metal contact. At elevated temperatures, solute diffusion increases, and the non-uniform distribution of hard phases, such as intermetallic compounds, causes the coating to be more susceptible to cutting by abrasive grains, resulting in furrow formation. Local adhesion results in material transfer and peeling. As shown in Figure 13b, the worn coating surface exhibits fine scratches and scattered oxide spots, indicating the coexistence of abrasive and oxidative wear mechanisms. Examining the elemental composition at points P3 and P4 in Table 7 shows that the Ni-to-Fe ratio is similar, and the γ-FeNi solid solution content is relatively high, enhancing toughness. The relatively high oxygen content promotes metal oxide formation. While the oxide layer offers partial protection, the carbon content is lower compared to the 800 mm/min coating. The non-uniform distribution of hard phases, such as metal carbides, continues to cause localized abrasive wear. As shown in Figure 13c, the worn coating surface is smooth, exhibiting microcracks and spalling, indicative of synergistic fatigue and oxidative wear mechanisms. Nb is highly enriched, tending to form brittle phases such as Fe_2_Nb, which can lead to cracking. The oxygen content is moderate, with the oxide film providing localized protection; however, the presence of the brittle phase disrupts the continuity of the oxide layer. This disruption accelerates localized wear once the oxide film is compromised. Figure 13d reveals uniform scratches and a continuous oxide film on the coating surface after friction and wear testing; oxidative wear is dominant, accompanied by adhesive wear simultaneously. The oxygen content increases substantially, potentially forming a continuous oxide film (e.g., Fe_3_O_4_ and Cr_2_O_3_). The oxide film isolates the friction pair, reducing abrasive particle wear. This transition is specific to NiAlNbTiV, as its eutectics are harder than those of CoCrFeMnNi [35] but more brittle than those of TiC-reinforced composites [33], creating a distinct wear behavior spectrum.

### 3.5. Corrosion Performance

**Figure 14:**
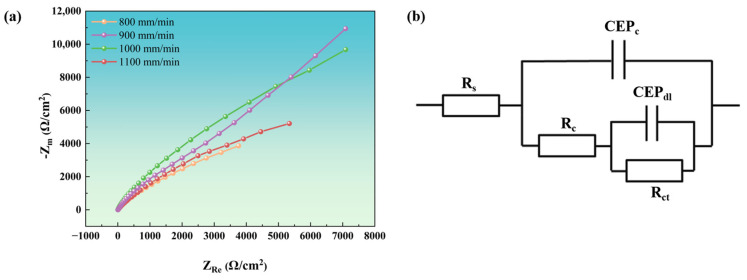
Electrochemical impedance spectra (EISs): (**a**) Nyquist plots for coatings deposited at various laser scanning speeds; (**b**) equivalent circuit diagram used for data fitting.

The diameter of the capacitive arc in the Nyquist diagram reflects variations in corrosion resistance among materials, with a larger diameter indicating superior corrosion resistance [41]. As shown in Figure 14a, the Nyquist plots of coatings deposited at different scanning rates exhibit incomplete impedance arcs, making direct determination of the arc diameter challenging. By fitting an equivalent circuit model and analyzing the raw data, the results in Figure 14b were obtained, enabling intuitive interpretation and analysis.

In Figure 14b, *R_s_* represents the solution resistance, reflecting the conductivity of the electrolyte. Higher values indicate poorer solution conductivity, which has a minimal impact on corrosion resistance [42]. The CEP, which stands for Constant Phase Element (CPE), is utilized to characterize non-ideal capacitive behavior. *C_c_* is the double-layer admittance constant. Smaller values indicate a more stable double layer, smoother electrode surface, and enhanced corrosion resistance. *n*_1_ is the non-ideal index of the double layer. Values closer to 1 indicate a more uniform surface and better corrosion resistance [43]. *R_c_* is the coating resistance. Higher values indicate a denser or more complete coating, enhancing resistance to corrosion [44]. *C_dl_* is the admittance constant of the coating film. Smaller values indicate a denser or thicker film with superior corrosion resistance. *n*_2_ is the non-ideal index of the coating. Values closer to 1 indicate better coating uniformity and corrosion resistance [45]. *R*_ct_ is the charge transfer resistance. Higher values indicate greater kinetic resistance to corrosion reactions, resulting in significantly enhanced corrosion resistance.

As shown in the fitting data in Table 8, the NiAlNbTiV coating exhibits optimal corrosion resistance at a laser scanning speed of 900 mm/min, followed by 1100 mm/min, 800 mm/min, and 1000 mm/min. This indicates that the relationship between laser cladding parameters and the corrosion resistance of the NiAlNbTiV coating is nonlinear. The coating produced at 900 mm/min exhibits the most superior corrosion resistance, attributed to its fine grain structure (which reduces intergranular corrosion) and minimal defects (that prevent electrolyte penetration). This optimal balance is contingent upon NiAlNbTiV’s capacity to form a stable Nb/Cr oxide film. In contrast, iron-rich HEAs [8] generally exhibit weaker protective effects from iron oxides.

The electrochemical impedance spectroscopy (EIS) testing results indicate that the coating produced at a scanning speed of 800 mm/min has a lower polarization resistance (R_c_). This can be attributed to the presence of coarse eutectic structures within the coating, which reduce its density, thereby diminishing its corrosion resistance. For the coating fabricated at a scanning speed of 1000 mm/min, the charge transfer resistance (R_ct_) reaches its lowest value. Rapid solidification during the laser cladding process results in a high grain-boundary density, which significantly increases the likelihood of grain-boundary corrosion. Notably, when the scanning speed increases to 1100 mm/min, the R_ct_ of the coating relatively increases, while the R_c_ decreases. This indicates that the formation of a nanostructure within the coating enhances the interfacial resistance at the interfaces. However, the increased density of cracks introduces additional pathways for medium penetration, ultimately reducing the overall corrosion resistance of the coating.

**Figure 15:**
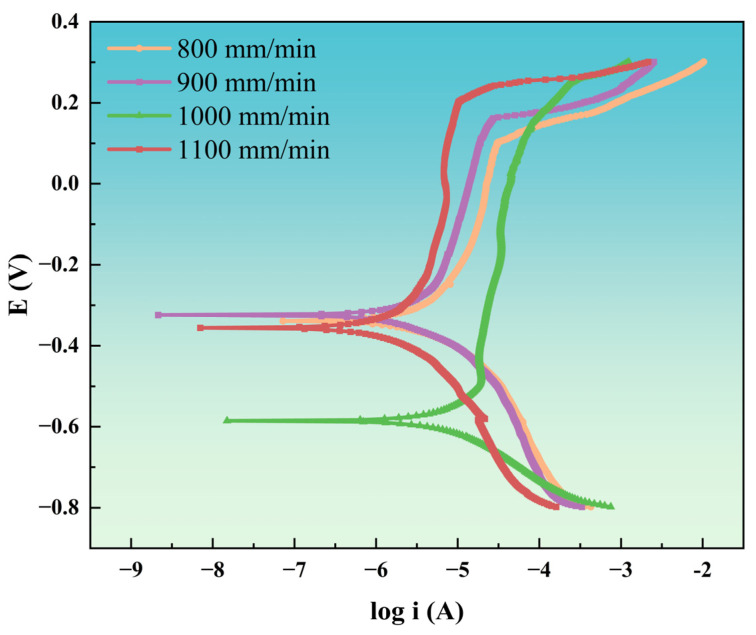
Polarization curves of coatings deposited at various laser scanning speeds.

To ensure experimental accuracy, polarization curve measurements were conducted. Coatings exhibiting a lower corrosion current density (*I*_corr_) and a more positive corrosion potential (*E*_corr_) demonstrate superior corrosion protection [46]. Figure 15 presents the polarization curves of coatings deposited at various laser scanning speeds, while Table 9 lists the corresponding fitting parameters for coatings immersed in 3.5 wt.% NaCl solution. As shown in Figure 15, the corrosion behavior of NiAlNbTiV coatings at different laser scanning speeds exhibits a similar trend: the corrosion current initially decreases to a minimum before increasing with further increases in corrosion potential. All coatings exhibit passivation behavior; however, coatings deposited at a laser scanning speed of 900 mm/min demonstrate a higher passivation-break potential and lower passivation current. Analysis of the polarization curve fitting data in Table 9 indicates that the coating deposited at 900 mm/min exhibits a lower *I*_corr_ and a more positive *E*_corr_. This phenomenon can be ascribed to several factors: the reduction in microgalvanic corrosion in fine-grained structures; the uniform oxide film, which helps to lower the corrosion rate; and the formation of a stable passivation film on the surface. These factors collectively contribute to effectively inhibiting anodic dissolution.

## 4. Conclusions

Microstructure and Phase Evolution: Laser scanning speed significantly regulates the phase composition and microstructure of NiAlNbTiV coatings. With increasing scanning speed, the cooling rate increases, promoting the formation of a γ-(Ni Fe) solid solution and enhancing the intensity of TiV phase peaks due to limited atomic diffusion. Microstructurally, higher scanning speeds induce grain refinement, with needle-like crystals becoming more uniformly distributed. However, excessive scanning speed (1100 mm/min) leads to increased point defects (28 defects/mm^2^) and the formation of cracks, attributed to insufficient powder melting and elevated residual stresses from rapid cooling.

Mechanical and Tribological Properties: The mechanical and tribological performance of the coatings is closely related to the scanning speed. Microhardness decreases with increasing scanning speed, from 606.2 HV at 800 mm/min to 522.4 HV at 1100 mm/min, due to reduced precipitation of hard intermetallic phases (e.g., Ni_3_Nb and Fe_2_Nb) under limited solute diffusion. Wear resistance exhibits a consistent trend with hardness: the 800 mm/min coating shows the best wear resistance (wear volume: 0.0117 mm^3^; mass loss: 0.5089 g) due to dense eutectic structures resisting abrasive wear. In contrast, higher scanning speeds (≥1000 mm/min) result in severe adhesive and fatigue wear, characterized by deep pits and interconnected grooves, caused by increased defects and reduced hardness.

Corrosion Resistance: The corrosion resistance of NiAlNbTiV coatings shows a non-linear relationship with scanning speed. The coating fabricated at 900 mm/min achieves optimal corrosion performance, with the lowest corrosion current density (1.656 μA·cm^−2^) and highest charge transfer resistance (60,780 Ω), benefiting from a fine-grained structure that reduces intergranular corrosion and a uniform oxide film (Nb/Cr oxides) inhibiting anodic dissolution. Coatings at 800 mm/min exhibit reduced corrosion resistance due to coarse eutectic structures, while those at 1000 mm/min suffer from enhanced grain-boundary corrosion. The overall corrosion performance of the coating at 1100 mm/min is affected by the electrolyte penetration induced by cracks.

## Figures and Tables

**Table 1:** Chemical compositions of 316 stainless steel and NiAlNbTiV powder (wt.%).

Element	Ni	Al	Nb	Ti	V	Mn	Fe	Si	Zn	Mo	C	Cr
316 stainless steel	12	-	-	-	-	2	Bal.	1	-	2.5	0.08	1.7
NiAlNbTiV powder	Bal.	6.05	20.3	10.9	11.2	-	-	0.09	0.18	-	0.01	-

**Table 2:** Number of coating point defects within 1 mm^2^.

Sample	Number of Point Defects/Pcs
Sample 800 mm/min	14
Sample 900 mm/min	18
Sample 1000 mm/min	21
Sample 1100 mm/min	28

**Table 3:** The content of elements on the coating surface varies with different scanning speeds (wt.%).

Sample	Ni	Al	Nb	Ti	V	Fe	Cr	Other
Sample 800 mm/min	34.60	2.05	7.54	3.63	3.18	34.84	12.25	Bal.
Sample 900 mm/min	43.09	3.24	11.71	7.51	6.39	19.92	7.03	Bal.
Sample 1000 mm/min	48.00	3.97	15.73	10.63	8.71	10.86	2.81	Bal.
Sample 1100 mm/min	48.99	5.84	16.94	11.43	10.01	8.05	2.01	Bal.

**Table 4:** The chemical composition at different positions in Figure 6a (wt %).

Position	Ni	Al	Nb	Ti	V	Fe	Cr	Other
Surface	21.42	1.98	7.78	4.44	4.18	36.53	10.03	Bal.
P1	18.90	01.65	11.95	04.61	03.80	36.14	09.23	Bal.
P2	28.60	02.22	03.28	04.70	05.24	32.41	08.64	Bal.
P3	15.00	01.39	21.77	04.78	03.23	34.06	08.32	Bal.

**Table 5:** Average hardness and growth rate of the coating with different laser speeds.

Sample	Mean Hardness Value/HV	Gaining Rate/%
Sample 800 mm/min	606.2	-
Sample 900 mm/min	564.8	−6.8%
Sample 1000 mm/min	536.0	−5.1%
Sample 1100 mm/min	522.4	−2.5%

**Table 6:** Friction and wear performance of the coating at various laser scanning speeds.

Sample	Wear Volume/mm^3^	Wear Quality/g
Sample 800 mm/min	0.011710	0.5089
Sample 900 mm/min	0.012951	0.5631
Sample 1000 mm/min	0.030026	1.3055
Sample 1100 mm/min	0.031958	1.3895

**Table 7:** The chemical compositions at different points in Figure 13 (wt.%).

Position	Ni	Al	Nb	Ti	V	Fe	Cr	O	Other
P1	29.77	02.25	09.01	04.92	04.37	30.64	07.82	08.78	Bal.
P2	25.24	02.56	05.90	06.78	05.60	33.08	09.31	09.92	Bal.
P3	34.38	02.51	06.53	04.67	04.55	32.18	08.12	05.92	Bal.
P4	26.16	02.47	06.26	04.55	05.24	34.41	09.59	10.25	Bal.
P5	29.21	02.06	17.43	05.64	04.72	26.37	06.21	06.72	Bal.
P6	31.59	02.72	04.59	08.02	06.35	27.39	08.34	10.06	Bal.
P7	30.28	02.63	07.03	05.05	04.09	33.93	08.64	07.05	Bal.
P8	21.31	01.78	06.02	04.96	03.33	38.37	09.62	13.83	Bal.

**Table 8:** EIS fitting parameters for coatings deposited at various laser scanning speeds in 3.5 wt.% NaCl solution.

Sample	R_s_/Ω	C_c_/μF·cm^−2^	n_1_	R_c_/Ω	C_dl_/μF·cm^−2^	n_2_	R_ct_/Ω	X
Sample 800 mm/min	3.769	180.1	0.691	2564	94.58	0.908	15,750	0.000601
Sample 900 mm/min	3.809	61.52	0.858	12480	66.50	0.922	60,780	0.000614
Sample 1000 mm/min	2.864	77.48	0.846	8902	73.67	0.857	42,460	0.001770
Sample 1100 mm/min	3.628	106.8	0.793	4709	70.99	0.920	57,570	0.000146

**Table 9:** Polarization curve fitting parameters for coatings deposited at various laser scanning speeds immersed in 3.5 wt.% NaCl solution.

Sample	I_corr_ /μA·cm^−2^	E_corr_/V
Sample 800 mm/min	2.000	−0.336
Sample 900 mm/min	1.656	−0.319
Sample 1000 mm/min	4.677	−0.584
Sample 1100 mm/min	1.724	−0.356

## Data Availability

The original contributions presented in this study are included in the article. Further inquiries can be directed to the corresponding authors.
